# Supplementary material for: Machine learning approaches identify immunologic signatures of total and intact HIV DNA during long-term antiretroviral therapy
Source: eLife. 2024 Sep 9;13:RP94899. doi: 10.7554/eLife.94899 (PMC11383529; doi:10.7554/eLife.94899)
Supplement: Supplementary file 1. — (a) Intact, total reservoir frequency, and %intact for demographic subgroups. (b) Immune subsets characteristics. (c) Host features correlate with HIV reservoir characteristics. (d) People with HIV (PWH) immune features correlate with years of antiretroviral therapy (ART). (e) Multicolinearity analysis for variables used in models to predict immunophenotypes. (f) Adjusted R2 scores and differences in adjusted R2 for leave-one-covariate-out (LOCO) analysis for the model that contains total reservoir frequency. (g) Adjusted R2 scores and differences in adjusted R2 for LOCO analysis for the model that contains intact reservoir frequency. (h) Adjusted R2 scores and differences in adjusted R2 for LOCO analysis for the model that contains percent intact. (i) Host features classify PWH with respect to HIV reservoir characteristics. (j) Training procedure for classification (regression). (k) Ranges of hyperparameters values that we used to perform grid search for classification and regression. [file elife-94899-supp1.pdf]

Supplementary File 1

Supplementary File 1a. *Intact, Total reservoir frequency and % intact for demographic subgroups.*
We report medians and [Q1, Q3] for total reservoir frequency, intact reservoir frequency (per million CD4 T cells), and percent intact. Mann-Whitney U test was used to compute p-values for gender and Kruskal-Wallis H-test for race.

Group	Size	Intact HIV DNA	Total HIV DNA	% intact HIV DNA
**Male**	88	63.5 [18.75 146.25]	681 [198 1433.75]	8.73 [4.9 20.58]
**Female**	27	41 [19, 92.5]	489 [317.5, 637]	8.64 [4.86, 15.56]
**p-value**		0.4521	0.1937	0.9451
**Black**	69	43 [19, 113]	502 [181, 1246]	9.77 [4.64, 16.4]
**White**	43	69 [26 191.5]	605 [289.5 1415.5]	8 [5.31 20.81]
**Other**	3	67 [41, 520]	1055 [720, 1165,]	6.39 [5.20, 41.37]
**p-value**		0.5079	0.4916	0.9914

Supplementary File 1b. *Immune subsets characteristics*. We report medians, 25% and 75% percentiles for every immune variable.

Immune marker	Med	[Q1, Q3]	Immune marker	Med	[Q1, Q3]
%CD4 T	52.90	[43.85, 59.60]	%CD27+ CD4 T	86.20	[79.15, 90.10]
%CD38+ CD4 T	46.60	[39.65, 54.30]	%CD127+ CD4 T	97.10	[95.20, 98.30]
%HLA-DR+ CD4 T	12.30	[9.76, 16.40]	%KLRG1+ CD4 T	17.20	[10.85, 26.25]
%NKG2A+ CD4 T	0.10	[0.06, 0.20]	%PD-1+ CD4 T	45.40	[39.70, 54.25]
%Tcm CD4 T	49.90	[42.30, 56.65]	%Tn CD4 T	30.40	[22.10, 39.90]
%Teff CD4 T	1.73	[0.89, 3.50]	%Tem CD4 T	15.70	[10.95, 19.70]
%CD38-/HLA-DR+ CD4 T	8.16	[6.22, 11.00]	%CD38+/HLA-DR+ CD4 T	4.37	[3.16, 5.94]
%CD38+/HLA-DR- CD4 T	40.20	[32.90, 47.75]	%KLRG1-/CD27+ CD4 T	74.40	[67.30, 83.05]
%KLRG1+/CD27+ CD4 T	6.70	[3.67, 11.65]	%KLRG1+/CD27- CD4 T	7.68	[4.54, 13.40]
%KLRG1-/CD27- CD4 T	6.01	[4.43, 8.04]	%KLRG1-/PD-1+ CD4 T	32.80	[27.00, 37.95]
%KLRG1+/PD-1+ CD4 T	12.60	[7.46, 17.30]	%KLRG1+/PD-1- CD4 T	4.79	[3.12, 8.63]
%KLRG1-/PD-1- CD4 T	49.30	[38.35, 55.90]	%PD-1-/CCR7+ CD4 T	50.90	[40.50, 56.35]
%PD-1+/CCR7+ CD4 T	29.70	[25.00, 35.05]	%PD-1+/CCR7- CD4 T	14.60	[10.40, 18.90]
%PD-1-/CCR7- CD4 T	3.77	[2.56, 5.25]	%CD8 T	40.30	[33.35, 47.45]
%CD27+ CD8 T	65.40	[54.05, 75.30]	%CD38+ CD8 T	35.70	[26.05, 45.45]
%CD127+ CD8 T	99.20	[98.40, 99.60]	%HLA-DR+ CD8 T	25.70	[18.75, 36.05]
%KLRG1+ CD8 T	62.10	[47.40, 73.40]	%PD-1+ CD8 T	37.50	[26.10, 47.50]
%Tn CD8 T	30.40	[21.60, 41.50]	%Teff CD8 T	26.90	[20.35, 36.70]
%Tem CD8 T	19.00	[13.00, 27.15]	%CD38-/HLA-DR+ CD8 T	16.10	[10.55, 22.75]
%CD38+/HLA-DR+ CD8 T	8.43	[5.86, 13.45]	%CD38+/HLA-DR- CD8 T	19.60	[14.45, 31.15]
%KLRG1-/CD27+ CD8 T	35.00	[23.45, 48.80]	%KLRG1+/CD27+ CD8 T	25.90	[19.35, 33.45]
%KLRG1+/CD27- CD8 T	30.30	[21.45, 43.40]	%KLRG1-/CD27- CD8 T	2.42	[1.58, 4.57]
%KLRG1-/PD-1+ CD8 T	7.40	[5.17, 10.95]	%KLRG1+/PD-1+ CD8 T	27.80	[18.35, 36.75]
%KLRG1+/PD-1- CD8 T	30.20	[23.35, 38.25]	%Tcm CD8 T	14.60	[10.30, 22.00]
%KLRG1-/PD-1- CD8 T	27.70	[17.75, 43.70]	%PD-1-/CCR7+ CD8 T	30.70	[22.45, 42.95]
%PD-1+/CCR7+ CD8 T	13.30	[10.10, 16.70]	%PD-1+/CCR7- CD8 T	22.00	[14.85, 27.50]
%PD-1-/CCR7- CD8 T	28.30	[18.20, 36.40]	%NK	6.37	[3.98, 10.50]
%NKG2A+ NK	31.60	[21.40, 49.85]	%NKG2A-/CD16+ NK	52.20	[34.25, 64.90]
%NKG2A+/CD16+ NK	16.50	[10.21, 24.35]	%NKG2A+/CD16- NK	13.10	[8.26, 23.75]
%NKG2A-/CD16- NK	13.80	[9.26, 19.15]	%NKT	4.33	[2.33, 6.56]
%NKG2A+ NKT	9.31	[4.79, 19.70]	%NKG2A-/CD16+ NKT	6.31	[1.66, 12.95]
%NKG2A+/CD16+ NKT	0.43	[0.14, 1.23]	%NKG2A+/CD16- NKT	8.48	[4.57, 17.65]
%NKG2A-/CD16- NKT	77.40	[65.85, 89.35]	%CD38-/HLA-DR+ Tcm CD4 T	9.93	[7.52, 12.25]
%CD38+/HLA-DR+ Tcm CD4 T	4.71	[3.13, 6.30]	%CD38+/HLA-DR- Tcm CD4 T	22.90	[17.00, 28.45]
%CD38-/HLA-DR- Tcm CD4 T	61.80	[55.20, 66.75]	%PD-1+ Tcm CD4 T	58.90	[52.55, 65.80]
%CD38-/HLA-DR+ Tn CD4 T	0.66	[0.30, 1.12]	%CD38+/HLA-DR+ Tn CD4 T	7.29	[5.21, 9.38]
%CD38+/HLA-DR- Tn CD4 T	81.30	[76.20, 87.55]	%CD38-/HLA-DR- Tn CD4 T	8.52	[5.80, 14.20]
%PD-1+ Tn CD4 T	7.26	[4.40, 12.65]	%CD38-/HLA-DR+ Teff CD4 T	17.50	[11.20, 25.75]
%CD38+/HLA-DR+ Teff CD4 T	19.00	[10.75, 28.15]	%CD38+/HLA-DR- Teff CD4 T	16.00	[11.25, 27.15]
%CD38-/HLA-DR- Teff CD4 T	38.50	[27.40, 50.50]	%PD-1+ Teff CD4 T	56.40	[39.30, 72.25]
%CD38-/HLA-DR+ Tem CD4 T	27.40	[22.45, 33.80]	%CD38+/HLA-DR+ Tem CD4 T	7.31	[5.31, 10.10]
%CD38+/HLA-DR- Tem CD4 T	4.77	[3.49, 6.96]	%CD38-/HLA-DR- Tem CD4 T	57.00	[50.75, 65.80]
%PD-1+ Tem CD4 T	82.20	[76.00, 86.55]	%CD38-/HLA-DR+ Tcm CD8 T	22.10	[16.70, 28.20]
%CD38+/HLA-DR+ Tcm CD8 T	7.11	[4.23, 12.10]	%CD38+/HLA-DR- Tcm CD8 T	5.28	[3.71, 7.18]
%CD38-/HLA-DR- Tcm CD8 T	62.70	[51.65, 72.10]	%PD-1+ Tcm CD8 T	63.60	[53.80, 71.10]
%CD38-/HLA-DR+ Tn CD8 T	6.02	[3.29, 9.82]	%CD38+/HLA-DR+ Tn CD8 T	3.68	[2.42, 5.36]
%CD38+/HLA-DR- Tn CD8 T	45.40	[31.70, 57.25]	%CD38-/HLA-DR- Tn CD8 T	43.00	[31.75, 54.45]
%PD-1+ Tn CD8 T	15.00	[9.96, 24.35]	%CD38-/HLA-DR+ Teff CD8 T	27.80	[21.55, 37.25]
%CD38+/HLA-DR+ Teff CD8 T	16.60	[11.35, 25.95]	%CD38+/HLA-DR- Teff CD8 T	12.40	[7.18, 18.45]
%CD38-/HLA-DR- Teff CD8 T	39.20	[26.30, 47.20]	%PD-1+ Teff CD8 T	33.90	[24.10, 41.85]
%CD38-/HLA-DR+ Tem CD8 T	29.70	[21.55, 36.05]	%CD38+/HLA-DR+ Tem CD8 T	13.80	[9.31, 21.10]
%CD38+/HLA-DR- Tem CD8 T	5.31	[3.27, 9.88]	%CD38-/HLA-DR- Tem CD8 T	46.30	[35.30, 56.80]
%PD-1+ Tem CD8 T	62.40	[49.70, 71.80]	%IL-2+ CD8 T	0.00	[-0.03, 0.03]
%IL-2+ CD4 T	0.02	[0.01, 0.04]	%TNFα+ CD4 T	0.04	[0.01, 0.06]
%TNFα+ CD8 T	0.04	[0.01, 0.12]	%IFNγ+ CD4 T	0.02	[0.00, 0.04]
%IFNγ+ CD8 T	0.05	[0.01, 0.21]	%CD107+ CD4 T	0.02	[0.01, 0.04]

%CD107+ CD8 T	0.23	[0.12, 0.55]	%CD107+IFNγ+IL-2-TNFα- CD4 T	0.00	[0.00, 0.00]
%CD107+IFNγ-IL-2+TNF- CD4 T	0.00	[0.00, 0.00]	%CD107+IFNγ-IL-2-TNFα+ CD4 T	0.00	[0.00, 0.00]
%CD107+IFNγ-IL-2-TNFα- CD4 T	0.01	[0.01, 0.02]	%CD107-IFNγ+IL-2+TNFα- CD4 T	0.00	[0.00, 0.00]
%CD107-IFNγ+IL-2-TNFα+ CD4 T	0.00	[-0.01, 0.01]	%CD107-IFNγ+IL-2-TNFα- CD4 T	0.00	[-0.01, 0.00]
%CD107-IFNγ-IL-2+TNFα+ CD4 T	0.01	[0.01, 0.02]	%CD107-IFNγ-IL-2+TNFα- CD4 T	0.00	[-0.01, 0.00]
%CD107-IFNγ-IL-2-TNFα+ CD4 T	0.01	[0.00, 0.02]	%CD107+IFNγ+IL-2-TNFα- CD8T	0.03	[0.01, 0.07]
%CD107+IFNγ-IL-2+TNF- CD8 T	0.00	[0.00, 0.00]	%CD107+IFNγ-IL-2-TNFα+ CD8 T	0.01	[0.00, 0.03]
%CD107+IFNγ-IL-2-TNFα- CD8 T	0.14	[0.08, 0.30]	%CD107-IFNγ+IL-2+TNFα- CD8 T	0.00	[0.00, 0.00]
%CD107-IFNγ+IL-2-TNFα+ CD8 T	0.00	[0.00, 0.01]	%CD107-IFNγ+IL-2-TNFα- CD8 T	0.00	[-0.01, 0.01]
%CD107-IFNγ-IL-2+TNFα+ CD8 T	0.00	[0.00, 0.00]	%CD107-IFNγ-IL-2+TNFα- CD8 T	-0.01	[-0.04, 0.00]
%CD107-IFNγ-IL-2-TNFα+ CD8 T	0.00	[0.00, 0.00]			

Supplementary File 1c. *Host features correlate with HIV reservoir characteristics.*
The abundance of 133 immune cell populations was determined by flow cytometry and the HIV reservoir was quantified by intact proviral DNA assay for a cohort of 115 people with HIV (PWH). Each abundance and clinical and demographic variable was correlated with total HIV reservoir frequency, intact reservoir frequency, and the percentage of intact proviruses. Spearman correlation coefficients are shown for 69 variables that were correlated (p<0.05) with one or more reservoir characteristics. Each feature/subset is ranked by the absolute value of the correlation coefficient for the total reservoir frequency. Variables that had significant p-values (<0.05) after Benjamini–Hochberg correction for multiple comparisons (144 comparisons in our case) with one of the characteristics are shown in bold. For years of ART, correlations are computed based on 108 participants, excluding participants with missing years of ART values. For CD4 Nadir, correlations are computed based on 114 patients, excluding participants with the missing CD4 Nadir value.

Variable	Total HIV DNA	Intact HIV DNA	% intact HIV DNA	Variable	Total HIV DNA	Intact HIV DNA	% intact HIV DNA
%CD8 T	**0.4052**	**0.3562**	0.0068	%CD38+/HLA-DR- Tcm CD8 T	-0.2169	-0.0182	0.1970
%CD38+/HLA-DR- CD4 T	**-0.3891**	-0.1098	0.2664	%CD107-IFNγ+IL-2-TNFα- CD4 T	-0.2154	-0.1633	-0.0599
%KLRG1-/PD-1- CD4 T	**-0.3808**	-0.1289	0.2334	%KLRG1-/CD27+ CD8 T	-0.2112	-0.1894	-0.0021
%Tn CD4 T	**-0.3802**	-0.2031	0.1714	%KLRG1+/PD-1+ CD8 T	0.2089	0.1922	0.0473
%NKG2A+ CD4 T	**0.3618**	0.2904	0.0179	%CD38+/HLA-DR- Tn CD4 T	-0.2066	0.0140	0.1909
%PD-1-/CCR7+ CD4 T	**-0.3590**	-0.1082	0.2283	%PD-1+/CCR7- CD4 T	0.1971	0.0410	-0.1338
%CD4 T	**-0.3564**	**-0.3195**	-0.0079	%CD38-/HLA-DR+ CD8 T	0.1947	0.0572	-0.0860
%Tcm CD8 T	**0.3466**	0.1752	-0.1814	%KLRG1+ CD8 T	0.1887	0.1709	0.0048
%CD38+ CD4 T	**-0.3366**	-0.0611	0.2829	%KLRG1-/PD-1+ CD4 T	0.1877	0.0818	-0.0963
%PD-1-/CCR7- CD4 T	**0.3300**	0.1824	-0.0938	%NKG2A-/CD16+ NK	0.1855	0.0433	-0.1222
%CD38+/HLA-DR- CD8 T	**-0.3267**	-0.0837	0.2636	%KLRG1+/PD-1- CD4 T	0.1849	0.0616	-0.1388
%PD-1+ CD4 T	**0.3222**	0.0664	-0.2470	%CD38+/HLA-DR+ Tem CD4 T	0.1798	0.2668	0.1961
Age	**0.3172**	0.1669	-0.1471	%KLRG1+ CD4 T	0.1771	0.0002	-0.2034
CD4 Nadir	**-0.3164**	-0.1512	0.1927	%KLRG1+/PD-1+ CD4 T	0.1650	-0.0392	-0.2355
%PD-1+ Tn CD4 T	**0.3119**	0.1246	-0.0962	Years before ART < 1	-0.1645	0.0527	0.2513
%Tn CD8 T	**-0.3028**	-0.2697	0.0154	% NKT	0.1616	-0.0223	-0.1942
Years of ART	**0.3062**	-0.0688	**-0.4523**	%CD38-/HLA-DR+ Tn CD4 T	0.1612	-0.0675	-0.2160
%PD-1+/CCR7+ CD8 T	**0.2926**	0.1361	-0.1254	%PD-1+/CCR7- CD8 T	0.1352	0.1899	0.1057
%CD38-/HLA-DR+ CD4 T	**0.2849**	0.0920	-0.1500	%CD38-/HLA-DR- Tn CD8 T	0.1349	-0.0452	-0.2308
%KLRG1-/PD-1- CD8 T	**-0.2757**	-0.2182	0.0162	%CD38-/HLA-DR- Tcm CD8 T	0.1302	-0.0048	-0.2000
%PD-1+ Tn CD8 T	**0.2738**	0.1411	-0.0705	Recent CD4 count	-0.1302	-0.1944	-0.0970
%CD38+/HLA-DR- Tn CD8 T	**-0.2676**	-0.0610	0.2119	%CD127+ CD4 T	-0.1298	**-0.3160**	-0.2539
%PD-1+/CCR7+ CD4 T	**0.2665**	0.0859	-0.1673	Years before ART = NA	0.1183	-0.0312	-0.1950
%KLRG1+/CD27+ CD8 T	**0.2606**	0.2212	0.0319	%CD127+ CD8 T	0.1136	-0.1217	-0.2458
%CD27+ CD4 T	**-0.2602**	-0.0531	0.1767	%CD38+/HLA-DR+ Tcm CD8 T	-0.0920	0.0701	0.2340
%KLRG1-/CD27+ CD4 T	**-0.2575**	-0.0464	0.2171	%CD107-IFNγ-IL-2+TNFα+ CD8 T	0.0909	-0.0499	-0.2270
%HLA-DR+ CD4 T	**0.2565**	0.1110	-0.0970	%CD38+/HLA-DR- Teff CD8 T	-0.0833	0.1328	0.2462
%PD-1+ CD8 T	**0.2541**	0.1969	0.0035	%CD38+/HLA-DR- Tem CD8 T	-0.0660	0.1177	0.1958
%CD38+ CD8 T	**-0.2418**	0.0109	**0.3114**	%CD107-IFNγ-IL-2+TNFα+ CD4 T	0.0568	-0.1775	**-0.3223**
%CD38-/HLA-DR+ Tn CD8 T	**0.2404**	0.0738	-0.1346	%IL-2+ CD4 T	-0.0515	-0.1936	-0.1868
%Tem CD4 T	**0.2402**	0.0888	-0.1669	%CD38+/HLA-DR+ Tem CD8 T	0.0510	0.1694	0.2107
%PD-1-/CCR7+ CD8 T	-0.2321	-0.2173	-0.0237	%CD38+/HLA-DR+ CD8 T	0.0461	0.1660	0.1865
%CD38-/HLA-DR- Tem CD4 T	-0.2302	-0.2365	-0.1349	%CD107-IFNγ+IL-2+TNFα- CD4 T	0.0285	-0.2455	**-0.3265**
%KLRG1+/CD27- CD4 T	0.2271	0.0207	-0.1935	%CD38+/HLA-DR- Tem CD4 T	-0.0134	0.1551	0.2013
%Tcm CD4 T	0.2262	0.1745	-0.0492				

Supplementary File 1d. *PWH immune features correlate with years of ART.*
The abundance of 133 immune cell populations was determined by flow cytometry and the HIV reservoir was quantified by intact proviral DNA assay for a cohort of 115 people with HIV (PWH). Each abundance and clinical and demographic variable was correlated with years of therapy for all participants (middle column) and for participants who had been on therapy for less than 10 years (right column). Spearman correlation coefficients are shown for all variables that were correlated ($p<0.05$) after Benjamini-Hochberg correction with years of treatment for all participants or for participants that were on ART for less than 10 years. Variables that had significant p-value (<0.05) after correction for multiple comparisons are shown in bold. Correlation is computed based on 108 PWH for all participants and on 60 PWH for PWH who were on therapy for less than 10 years). Computations exclude PWH with missing years of ART values. For CD4 Nadir, correlations are computed excluding the participant with missing CD4 Nadir value.

Variable	Spearman correlation coefficient, All participants	Spearman correlation coefficient, Participants with years of ART< 10 years
Age	**0.4550**	0.2629
%CD38+/HLA-DR- CD8 T	**-0.4132**	-0.3788
%CD38+/HLA-DR- Tn CD8 T	**-0.4035**	-0.3075
%CD38+ CD8 T	**-0.3770**	-0.3025
%Tcm CD8 T	**0.3684**	0.1595
%CD38-/HLA-DR- Tn CD8 T	**0.3580**	0.2222
%CD38+/HLA-DR- Tcm CD8 T	**-0.3138**	-0.2666
CD4 Nadir	**-0.3078**	0.1265
%CD107a+IFNγ-IL-2+TNF- CD8 T	**0.2903**	0.1728

Supplementary File 1e. *Multicolinearity analysis for variables used in models to predict immunophenotypes.*
Variance Inflation Factor (VIF) for every variable is computed for models that fit immunophenotypes as a target and are based on clinical, demographic information and HIV characteristics (total, intact reservoir frequency or percent intact) as features. Note that here "Years before ART" is a continuous variable that we used for VIF computations only. In our other analysis we categorize this variable and use "Years before ART=NA", "Years before ART< 1", and "Years before ART≥ 1" instead. We excluded 3 participants with race indicated as "Other" to avoid near-binary categorical variables in VIF computation.

Total HIV DNA model		Intact HIV DNA model		% intact HIV DNA model	
**Variable**	**VIF value**	**Variable**	**VIF value**	**Variable**	**VIF value**
Total HIV DNA	1.2545	Intact HIV DNA	1.0517	% intact HIV DNA	1.3469
Age	1.7426	Age	1.7243	Age	1.7220
Sex	1.1948	Sex	1.1616	Sex	1.1795
Race	1.5080	Race	1.4577	Race	1.6348
Years of ART	1.3048	Years of ART	1.3004	Years of ART	1.3181
CD4 nadir	1.3188	CD4 nadir	1.2875	CD4 nadir	1.3773
Recent CD4 count	1.3314	Recent CD4 count	1.3374	Recent CD4 count	1.3134
Years before ART	1.1367	Years before ART	1.1138	Years before ART	1.1611

Supplementary File 1f. *Adjusted R^2 scores and differences in adjusted R^2 for LOCO analysis for the model that contains total reservoir frequency.* The first column contains all immunophenotypes and is a target for the regression model. The second column contains adjusted R^2 of the linear regression model with features such as total reservoir frequency, age, biological sex, race, years of treatment, CD4 nadir, recent CD4 count, and years of HIV before treatment (=NA, < 1, ≥ 1). The next seven columns show differences in adjusted R^2 score (ΔR^2) after removing a specific feature. Participants with missing years of ART values are excluded from this analysis. The missing value of the CD4 nadir for one participant is imputed.

	Adj. R^2	ΔR^2, difference in adjustable R^2 after dropping a covariate							
Model / Immunophenotype	Include all	Total HIV DNA	Age	Sex	Years of ART	CD4 nadir	Recent CD4 count	Years before ART	Race
%CD4 T	0.2583	0.0443	0.0024	0.0365	-0.0006	0.0079	0.1374	-0.0184	-0.0153
%CD27+ CD4 T	0.1031	0.0050	-0.0015	0.0191	-0.0017	-0.0028	0.0282	0.0026	0.0651
%CD38+ CD4 T	0.0970	-0.0011	0.0390	0.0201	-0.0092	0.0315	0.0017	-0.0241	0.0233
%CD127+ CD4 T	-0.0229	-0.0057	0.0235	-0.0076	-0.0105	0.0004	0.0085	-0.0299	-0.0203
%HLA-DR+ CD4 T	0.1862	0.0816	-0.0083	0.0294	-0.0061	-0.0043	0.0236	-0.0117	0.0161
%KLRG1+ CD4 T	0.0638	-0.0044	-0.0047	-0.0089	-0.0076	0.0029	0.0049	0.0135	0.0154
%NKG2A+ CD4 T	0.1082	0.0820	0.0303	-0.0043	-0.0077	-0.0020	-0.0034	-0.0214	-0.0097
%PD-1+ CD4 T	0.1175	0.0304	0.0017	0.0152	-0.0041	0.0025	0.0189	-0.0270	0.0019
%Tcm CD4 T	0.0192	-0.0101	0.0191	-0.0083	-0.0086	-0.0010	-0.0101	-0.0110	-0.0186
%Tn CD4 T	0.0960	0.0044	0.0343	0.0102	-0.0072	-0.0071	0.0249	-0.0276	-0.0179
%Teff CD4 T	-0.0298	-0.0013	-0.0073	-0.0033	-0.0090	0.0010	-0.0041	-0.0193	0.0111
%Tem CD4 T	0.0585	0.0046	-0.0072	0.0026	-0.0084	-0.0098	0.0450	-0.0060	0.0105
%CD38-/HLA-DR+ CD4 T	0.1711	0.0546	0.0004	0.0451	0.0052	0.0070	-0.0041	0.0099	0.0372
%CD38+/HLA-DR+ CD4 T	0.1159	0.0655	-0.0030	0.0012	-0.0076	-0.0089	0.0544	-0.0270	-0.0175
%CD38+/HLA-DR- CD4 T	0.1603	0.0246	0.0290	0.0351	-0.0077	0.0269	-0.0085	-0.0217	0.0383
%KLRG1-/CD27+ CD4 T	0.0438	-0.0090	-0.0078	-0.0094	-0.0056	0.0045	0.0198	0.0002	-0.0180
%KLRG1+/CD27+ CD4 T	0.2228	-0.0001	-0.0071	0.0153	-0.0080	0.0532	-0.0077	-0.0217	0.1907
%KLRG1+/CD27- CD4 T	0.0351	-0.0100	0.0064	0.0064	-0.0044	-0.0004	0.0117	0.0287	0.0109
%KLRG1-/CD27- CD4 T	0.2694	0.1007	-0.0005	0.0139	-0.0042	-0.0068	0.0213	-0.0046	0.0948
%KLRG1-/PD-1+ CD4 T	0.1404	0.0904	-0.0042	-0.0021	-0.0089	-0.0053	0.0005	0.0089	0.0346
%KLRG1+/PD-1+ CD4 T	0.0112	-0.0013	-0.0068	0.0024	-0.0003	-0.0048	0.0022	0.0200	-0.0254
%KLRG1+/PD-1- CD4 T	0.1251	-0.0087	-0.0053	0.0304	-0.0078	0.0066	-0.0006	-0.0134	0.0893
%KLRG1-/PD-1- CD4 T	0.1403	0.0178	0.0064	-0.0079	-0.0070	0.0170	0.0299	-0.0216	-0.0252
%PD-1-/CCR7+ CD4 T	0.1334	0.0369	0.0092	0.0089	-0.0066	-0.0038	0.0321	-0.0251	0.0135
%PD-1+/CCR7+ CD4 T	0.0690	0.0157	0.0073	-0.0067	-0.0061	0.0188	-0.0083	-0.0079	-0.0197
%PD-1+/CCR7- CD4 T	0.0433	0.0040	-0.0098	0.0164	-0.0085	-0.0096	0.0284	-0.0052	0.0045
%PD-1-/CCR7- CD4 T	0.0038	0.0072	0.0134	-0.0100	-0.0076	-0.0029	0.0276	-0.0181	0.0040
%CD8 T	0.2546	0.0603	-0.0077	0.0334	-0.0066	0.0068	0.1239	-0.0188	-0.0138
%CD27+ CD8 T	0.0584	0.0020	0.0364	0.0119	-0.0091	-0.0097	0.0243	-0.0058	0.0162
%CD38+ CD8 T	0.2543	-0.0068	0.0692	0.0085	-0.0062	0.0382	0.0806	-0.0005	-0.0191
%CD127+ CD8 T	-0.0073	-0.0073	-0.0099	-0.0075	-0.0037	0.0053	0.0000	-0.0168	0.0226
%HLA-DR+ CD8 T	-0.0012	0.0039	0.0033	-0.0104	-0.0103	-0.0067	0.0299	-0.0201	-0.0255
%KLRG1+ CD8 T	0.2143	-0.0082	0.0547	0.0041	-0.0067	-0.0039	-0.0017	-0.0176	0.0456
%PD-1+ CD8 T	0.2642	0.0358	0.0090	0.0010	-0.0071	-0.0073	-0.0048	-0.0222	0.0932
%Tn CD8 T	0.2975	-0.0052	0.1074	0.0081	-0.0070	-0.0073	0.0088	-0.0061	0.0343
%Teff CD8 T	-0.0151	-0.0089	0.0207	0.0237	0.0126	-0.0103	-0.0037	-0.0251	0.0106
%Tem CD8 T	0.2716	-0.0076	-0.0075	-0.0061	-0.0020	-0.0076	0.0118	-0.0186	0.2503
%CD38-/HLA-DR+ CD8 T	0.0074	-0.0061	0.0323	-0.0099	-0.0103	-0.0102	-0.0035	0.0015	-0.0256
%CD38+/HLA-DR+ CD8 T	0.0112	0.0069	-0.0099	-0.0091	-0.0100	0.0023	0.0509	-0.0301	-0.0184
%CD38+/HLA-DR- CD8 T	0.3161	-0.0044	0.0852	0.0236	-0.0038	0.0223	0.0228	0.0063	-0.0009
%KLRG1-/CD27+ CD8 T	0.2091	-0.0082	0.0484	0.0075	-0.0063	-0.0038	0.0037	-0.0153	0.0421
%KLRG1+/CD27+ CD8 T	0.3370	0.0190	0.0007	-0.0069	-0.0055	0.0097	0.0013	-0.0166	0.2342
%KLRG1+/CD27- CD8 T	0.0396	0.0025	0.0452	0.0089	-0.0097	-0.0098	0.0148	-0.0104	-0.0074
%KLRG1-/CD27- CD8 T	0.0770	-0.0091	0.0004	-0.0058	-0.0083	-0.0096	0.0147	-0.0243	0.0798
%KLRG1-/PD-1+ CD8 T	0.0189	0.0384	-0.0052	-0.0098	-0.0091	-0.0102	-0.0097	-0.0299	0.0340
%KLRG1+/PD-1+ CD8 T	0.3163	0.0130	0.0057	0.0017	-0.0070	-0.0069	-0.0025	-0.0206	0.1568
%KLRG1+/PD-1- CD8 T	-0.0235	0.0086	0.0497	-0.0063	-0.0064	-0.0046	-0.0083	-0.0219	-0.0302
%Tcm CD8 T	0.2293	0.0124	0.0817	-0.0076	0.0043	-0.0076	-0.0031	-0.0230	-0.0154

%KLRG1-/PD-1- CD8 T	0.2662	-0.0041	0.0740	0.0077	-0.0067	-0.0024	-0.0014	-0.0147	0.0375
%PD-1-/CCR7+ CD8 T	0.1968	-0.0032	0.0539	0.0226	-0.0035	-0.0075	0.0040	-0.0130	0.0190
%PD-1+/CCR7+ CD8 T	0.1735	0.0550	0.0300	-0.0011	-0.0076	0.0003	0.0133	-0.0252	-0.0145
%PD-1+/CCR7- CD8 T	0.2468	0.0035	-0.0063	-0.0034	-0.0078	-0.0061	0.0226	-0.0219	0.1493
%PD-1-/CCR7- CD8 T	-0.0373	0.0053	0.0244	0.0060	0.0023	-0.0104	-0.0033	-0.0173	-0.0119
% NK	0.1125	-0.0065	-0.0081	0.1126	-0.0063	0.0048	-0.0084	-0.0247	-0.0051
%NKG2A+ NK	0.0933	0.0035	-0.0053	0.1413	-0.0080	-0.0088	0.0130	-0.0264	-0.0165
%NKG2A-/CD16+ NK	0.0840	0.0297	-0.0071	0.0991	-0.0080	-0.0027	0.0271	-0.0262	-0.0148
%NKG2A+/CD16+ NK	-0.0016	-0.0095	0.0071	0.0554	-0.0103	-0.0096	-0.0104	-0.0259	-0.0296
%NKG2A+/CD16- NK	0.1141	0.0121	-0.0089	0.1223	-0.0046	-0.0050	0.0449	-0.0271	-0.0038
%NKG2A-/CD16- NK	-0.0205	0.0306	-0.0099	-0.0025	-0.0106	0.0065	0.0015	-0.0309	-0.0301
% NKT	-0.0062	0.0020	0.0002	-0.0105	-0.0099	-0.0088	0.0003	-0.0297	0.0564
%NKG2A+ NKT	-0.0306	-0.0060	-0.0104	0.0000	0.0248	-0.0099	-0.0098	-0.0314	-0.0192
%NKG2A-/CD16+ NKT	0.0151	0.0655	-0.0070	0.0127	-0.0074	-0.0077	-0.0086	-0.0264	-0.0178
%NKG2A+/CD16+ NKT	0.0394	0.0089	-0.0100	0.0468	0.0118	-0.0096	0.0140	0.0052	0.0017
%NKG2A+/CD16- NKT	-0.0391	-0.0082	-0.0105	-0.0057	0.0204	-0.0096	-0.0075	-0.0315	-0.0205
%NKG2A-/CD16- NKT	0.0722	0.0513	-0.0068	0.0266	0.0279	-0.0096	-0.0097	-0.0274	-0.0004
%CD38-/HLA-DR+ Tcm CD4 T	-0.0030	0.0104	-0.0102	0.0238	0.0046	-0.0005	-0.0103	-0.0254	-0.0014
%CD38+/HLA-DR+ Tcm CD4 T	0.0657	-0.0001	0.0020	-0.0031	-0.0044	-0.0024	0.0839	-0.0286	-0.0213
%CD38+/HLA-DR- Tcm CD4 T	0.0952	0.0071	-0.0030	-0.0034	-0.0090	0.0325	0.0299	-0.0276	0.0636
%CD38-/HLA-DR- Tcm CD4 T	0.1248	-0.0062	0.0012	-0.0090	-0.0091	0.0276	0.0680	-0.0264	0.0442
%PD-1+ Tcm CD4 T	0.0118	0.0225	-0.0098	-0.0102	-0.0089	0.0017	-0.0019	-0.0302	0.0069
%CD38-/HLA-DR+ Tn CD4 T	-0.0410	0.0206	-0.0080	0.0040	-0.0045	-0.0059	-0.0078	-0.0260	-0.0213
%CD38+/HLA-DR+ Tn CD4 T	-0.0505	-0.0065	-0.0105	-0.0107	-0.0097	-0.0106	0.0317	-0.0317	-0.0137
%CD38+/HLA-DR- Tn CD4 T	0.0408	0.0226	-0.0095	0.0273	-0.0094	0.0053	0.0224	-0.0285	0.0064
%CD38-/HLA-DR- Tn CD4 T	0.0616	0.0115	-0.0097	0.0341	-0.0093	0.0094	0.0772	-0.0276	-0.0104
%PD-1+ Tn CD4 T	0.0946	0.0970	-0.0085	0.0065	-0.0074	0.0011	-0.0072	-0.0254	0.0230
%CD38-/HLA-DR+ Teff CD4 T	0.0287	-0.0086	0.0171	0.0008	-0.0100	-0.0017	0.0122	-0.0125	0.0352
%CD38+/HLA-DR+ Teff CD4 T	0.1811	-0.0032	0.0474	-0.0080	0.0024	0.0106	0.0711	-0.0241	0.0464
%CD38+/HLA-DR- Teff CD4 T	0.1149	-0.0078	0.0454	-0.0064	-0.0090	-0.0089	0.0035	0.0016	0.0993
%CD38-/HLA-DR- Teff CD4 T	0.1315	0.0023	0.0462	-0.0089	-0.0055	0.0132	0.0289	-0.0265	0.0438
%PD-1+ Teff CD4 T	0.0153	-0.0022	-0.0046	0.0616	0.0091	-0.0077	0.0027	-0.0246	-0.0116
%CD38-/HLA-DR+ Tem CD4 T	0.0211	0.0126	-0.0041	0.0020	0.0123	0.0045	0.0083	0.0021	0.0096
%CD38+/HLA-DR+ Tem CD4 T	0.1387	0.1481	-0.0077	-0.0040	-0.0090	-0.0064	0.0186	-0.0200	-0.0172
%CD38+/HLA-DR- Tem CD4 T	-0.0344	-0.0091	-0.0088	-0.0083	-0.0106	0.0357	0.0169	0.0010	-0.0308
%CD38-/HLA-DR- Tem CD4 T	0.0779	0.1106	-0.0071	0.0007	0.0022	-0.0093	-0.0064	-0.0279	0.0054
%PD-1+ Tem CD4 T	-0.1111	-0.0115	-0.0050	-0.0108	-0.0093	-0.0116	-0.0116	-0.0340	-0.0273
%CD38-/HLA-DR+ Tcm CD8 T	-0.0878	-0.0112	-0.0087	-0.0079	-0.0101	-0.0107	-0.0063	-0.0325	-0.0232
%CD38+/HLA-DR+ Tcm CD8 T	0.0513	0.0153	0.0206	-0.0067	-0.0088	0.0086	0.0399	-0.0201	-0.0203
%CD38+/HLA-DR- Tcm CD8 T	0.0880	-0.0057	0.0342	0.0043	-0.0094	0.0495	0.0284	-0.0255	-0.0266
%CD38-/HLA-DR- Tcm CD8 T	0.0719	0.0021	0.0298	-0.0068	-0.0096	0.0164	0.0489	-0.0249	-0.0271
%PD-1+ Tcm CD8 T	-0.0177	-0.0038	-0.0065	-0.0009	-0.0090	-0.0104	0.0083	-0.0310	-0.0102
%CD38-/HLA-DR+ Tn CD8 T	0.0511	0.0455	0.0163	-0.0066	-0.0075	-0.0021	-0.0089	-0.0285	-0.0246
%CD38+/HLA-DR+ Tn CD8 T	-0.0188	0.0216	-0.0024	-0.0098	-0.0044	-0.0009	0.0257	-0.0305	-0.0295
%CD38+/HLA-DR- Tn CD8 T	0.2816	0.0145	0.0168	0.0247	-0.0051	0.0255	0.0751	-0.0209	0.0336
%CD38-/HLA-DR- Tn CD8 T	0.2317	-0.0079	0.0090	0.0168	-0.0059	0.0274	0.1329	-0.0223	0.0274
%PD-1+ Tn CD8 T	0.0747	0.0523	0.0190	0.0119	-0.0077	-0.0075	-0.0011	-0.0283	-0.0223
%CD38-/HLA-DR+ Teff CD8 T	0.0617	-0.0096	0.0031	-0.0047	0.0126	-0.0085	-0.0091	0.0011	0.0306
%CD38+/HLA-DR+ Teff CD8 T	-0.0083	-0.0044	-0.0046	0.0012	-0.0102	0.0004	0.0389	-0.0280	0.0003
%CD38+/HLA-DR- Teff CD8 T	0.1117	0.0032	0.0100	-0.0056	0.0071	0.0161	0.0178	0.0111	0.0278
%CD38-/HLA-DR- Teff CD8 T	0.0079	0.0048	-0.0070	0.0218	-0.0080	0.0140	0.0789	-0.0294	-0.0279
%PD-1+ Teff CD8 T	0.0093	-0.0035	-0.0057	-0.0074	-0.0099	-0.0093	0.0000	-0.0249	0.0604
%CD38-/HLA-DR+ Tem CD8 T	-0.0024	-0.0095	0.0033	-0.0021	-0.0020	-0.0099	-0.0104	0.0058	-0.0132
%CD38+/HLA-DR+ Tem CD8 T	-0.0234	0.0178	-0.0045	-0.0106	-0.0083	-0.0042	0.0334	-0.0310	-0.0304
%CD38+/HLA-DR- Tem CD8 T	0.0303	-0.0046	-0.0054	-0.0096	0.0046	-0.0015	0.0181	0.0005	-0.0015
%CD38-/HLA-DR- Tem CD8 T	-0.0335	0.0082	-0.0108	-0.0053	-0.0074	0.0029	0.0410	-0.0282	-0.0278
%PD-1+ Tem CD8 T	-0.0275	0.0265	-0.0085	0.0005	-0.0106	-0.0076	-0.0102	-0.0309	-0.0142
%IL-2+ CD8 T	-0.0375	0.0109	-0.0099	-0.0092	0.0091	-0.0108	-0.0092	-0.0303	0.0064

%IL-2+ CD4 T	0.0355	0.0529	-0.0051	0.0031	0.0119	-0.0015	-0.0090	-0.0166	-0.0128
%TNFa+ CD4 T	-0.0180	0.0065	-0.0106	0.0065	0.0256	-0.0099	0.0059	-0.0144	-0.0149
%TNFa+ CD8 T	-0.0562	0.0118	-0.0046	-0.0086	0.0152	-0.0041	0.0119	-0.0294	-0.0309
%IFNg+ CD4 T	-0.0646	-0.0003	-0.0099	-0.0067	-0.0085	-0.0110	-0.0055	-0.0254	-0.0133
%IFNg+ CD8 T	-0.0831	0.0019	-0.0096	-0.0110	-0.0039	-0.0100	0.0092	-0.0285	-0.0230
%CD107+ CD4 T	-0.0805	-0.0101	-0.0080	-0.0112	-0.0037	-0.0109	0.0090	-0.0263	-0.0263
%CD107+ CD8 T	-0.0856	0.0007	-0.0078	-0.0077	-0.0007	-0.0088	0.0098	-0.0332	-0.0311
%CD107+IFNg+IL-2-TNFa- CD4 T	-0.0835	-0.0105	-0.0076	-0.0097	-0.0098	-0.0111	0.0007	-0.0272	-0.0260
%CD107+IFNg-IL-2+TNF- CD4 T	-0.0745	-0.0108	-0.0107	-0.0109	-0.0107	-0.0075	0.0036	-0.0157	-0.0255
%CD107+IFNg-IL-2-TNFa+ CD4 T	-0.0750	-0.0097	-0.0112	-0.0100	0.0067	0.0115	-0.0030	-0.0170	-0.0284
%CD107+IFNg-IL-2-TNFa- CD4 T	-0.0386	-0.0064	-0.0001	-0.0098	0.0090	-0.0094	0.0339	-0.0318	-0.0249
%CD107-IFNg+IL-2+TNFa- CD4 T	-0.0170	0.0203	0.0030	0.0028	-0.0102	-0.0050	0.0214	-0.0262	-0.0130
%CD107-IFNg+IL-2-TNFa+ CD4 T	-0.0478	-0.0108	-0.0051	0.0295	-0.0068	-0.0029	0.0007	-0.0319	-0.0244
%CD107-IFNg+IL-2-TNFa- CD4 T	-0.0048	-0.0009	-0.0100	0.0054	-0.0052	-0.0104	-0.0023	-0.0304	-0.0055
%CD107-IFNg-IL-2+TNFa+ CD4 T	0.1865	0.0390	-0.0016	0.0255	0.0991	0.0104	0.0024	-0.0133	-0.0177
%CD107-IFNg-IL-2+TNFa- CD4 T	0.0568	0.0783	0.0015	0.0032	-0.0087	0.0079	-0.0073	-0.0222	-0.0152
%CD107-IFNg-IL-2-TNFa+ CD4 T	-0.0205	-0.0085	-0.0105	0.0024	0.0467	-0.0025	0.0011	-0.0100	-0.0184
%CD107+IFNg+IL-2-TNFa- CD8T	-0.0906	-0.0061	-0.0113	-0.0113	-0.0109	-0.0112	0.0061	-0.0301	-0.0221
%CD107+IFNg-IL-2+TNF- CD8 T	-0.0248	-0.0103	0.0132	-0.0107	0.0117	-0.0019	-0.0086	-0.0266	-0.0106
%CD107+IFNg-IL-2-TNFa+ CD8 T	-0.0177	0.0067	0.0014	0.0051	0.0033	0.0101	0.0005	-0.0285	-0.0218
%CD107+IFNg-IL-2-TNFa- CD8 T	-0.0361	-0.0035	-0.0048	0.0036	0.0063	-0.0091	0.0084	-0.0136	-0.0292
%CD107-IFNg+IL-2+TNFa- CD8 T	-0.0046	0.0323	0.0150	-0.0090	0.0069	-0.0104	0.0018	-0.0238	0.0026
%CD107-IFNg+IL-2-TNFa+ CD8 T	0.0194	0.0193	0.0068	-0.0102	0.0553	-0.0058	0.0312	-0.0293	-0.0262
%CD107-IFNg+IL-2-TNFa- CD8 T	-0.0374	0.0055	-0.0105	-0.0108	-0.0065	-0.0092	0.0036	-0.0301	0.0213
%CD107-IFNg-IL-2+TNFa+ CD8 T	0.0283	-0.0101	-0.0063	0.0087	0.0924	-0.0004	0.0149	-0.0269	-0.0066
%CD107-IFNg-IL-2+TNFa- CD8 T	-0.0558	-0.0022	-0.0102	-0.0100	-0.0109	-0.0084	-0.0081	-0.0278	-0.0006
%CD107-IFNg-IL-2-TNFa+ CD8 T	-0.0037	0.0017	-0.0094	-0.0092	0.0149	-0.0103	-0.0104	-0.0248	-0.0188

Supplementary File 1g. *Adjusted R^2 scores and differences in adjusted R^2 for LOCO analysis for the model that contains intact reservoir frequency.* The first column contains all immunophenotypes and is a target for the regression model. The second column contains adjusted R^2 of the linear regression model with features such as intact reservoir frequency, age, biological sex, race, years of treatment, CD4 nadir, recent CD4 count, and years of HIV before treatment (=NA, < 1, ≥ 1). The next seven columns show differences in adjusted R^2 score (ΔR^2) after removing a specific feature. Participants with missing years of ART values are excluded from this analysis. The missing value of the CD4 nadir for one participant is imputed.

	Adj. R^2	ΔR^2, difference in adjustable R^2 after dropping a covariate							
Immunophenotype \ Model	Include all	Intact HIV DNA	Age	Sex	Years of ART	CD4 nadir	Recent CD4 count	Years before ART	Race
%CD4 T	0.2423	0.0283	-0.0013	0.0266	0.0047	0.0204	0.1378	-0.0138	-0.0131
%CD27+ CD4 T	0.0896	-0.0086	0.0016	0.0264	0.0021	-0.0056	0.0325	0.0069	0.0708
%CD38+ CD4 T	0.0906	-0.0075	0.0482	0.0276	-0.0083	0.0373	-0.0021	-0.0235	0.0275
%CD127+ CD4 T	0.0550	0.0722	0.0202	-0.0074	-0.0095	-0.0028	0.0008	-0.0254	-0.0242
%HLA-DR+ CD4 T	0.1658	0.0612	-0.0072	0.0450	-0.0085	0.0059	0.0233	-0.0010	0.0286
%KLRG1+ CD4 T	0.0818	0.0136	-0.0043	-0.0085	-0.0076	-0.0003	0.0084	0.0084	0.0192
%NKG2A+ CD4 T	0.1013	0.0751	0.0221	0.0017	-0.0093	0.0112	-0.0041	-0.0130	0.0017
%PD-1+ CD4 T	0.0859	-0.0012	0.0072	0.0252	0.0007	0.0109	0.0229	-0.0271	0.0068
%Tcm CD4 T	0.0191	-0.0102	0.0202	-0.0080	-0.0084	-0.0006	-0.0101	-0.0111	-0.0187
%Tn CD4 T	0.0829	-0.0088	0.0421	0.0161	-0.0051	-0.0050	0.0290	-0.0277	-0.0173
%Teff CD4 T	-0.0378	-0.0093	-0.0058	-0.0005	-0.0100	-0.0021	-0.0030	-0.0169	0.0145
%Tem CD4 T	0.0448	-0.0092	-0.0053	0.0073	-0.0067	-0.0098	0.0504	-0.0024	0.0126
%CD38-/HLA-DR+ CD4 T	0.1345	0.0180	0.0058	0.0626	-0.0016	0.0201	-0.0033	0.0220	0.0482
%CD38+/HLA-DR+ CD4 T	0.1327	0.0823	-0.0052	0.0081	-0.0044	-0.0078	0.0497	-0.0243	-0.0094
%CD38+/HLA-DR- CD4 T	0.1300	-0.0057	0.0394	0.0484	-0.0054	0.0395	-0.0091	-0.0197	0.0452
%KLRG1-/CD27+ CD4 T	0.0473	-0.0055	-0.0066	-0.0088	-0.0040	0.0054	0.0249	0.0002	-0.0179
%KLRG1+/CD27+ CD4 T	0.2304	0.0074	-0.0068	0.0183	-0.0078	0.0457	-0.0074	-0.0226	0.1975
%KLRG1+/CD27- CD4 T	0.0426	-0.0026	0.0088	0.0084	-0.0032	0.0006	0.0158	0.0271	0.0133
%KLRG1-/CD27- CD4 T	0.2528	0.0842	-0.0044	0.0268	0.0015	0.0005	0.0204	-0.0152	0.1125
%KLRG1-/PD-1+ CD4 T	0.1170	0.0671	0.0001	0.0053	-0.0082	0.0051	0.0003	-0.0061	0.0500
%KLRG1+/PD-1+ CD4 T	0.0391	0.0266	-0.0063	0.0020	-0.0005	-0.0072	0.0064	0.0130	-0.0212
%KLRG1+/PD-1- CD4 T	0.1276	-0.0062	-0.0051	0.0315	-0.0078	0.0057	0.0004	-0.0143	0.0908
%KLRG1-/PD-1- CD4 T	0.1160	-0.0065	0.0123	-0.0059	-0.0043	0.0266	0.0353	-0.0195	-0.0264
%PD-1-/CCR7+ CD4 T	0.0957	-0.0008	0.0170	0.0182	-0.0028	0.0025	0.0378	-0.0238	0.0195
%PD-1+/CCR7+ CD4 T	0.0520	-0.0013	0.0123	-0.0040	-0.0031	0.0294	-0.0078	-0.0135	-0.0172
%PD-1+/CCR7- CD4 T	0.0305	-0.0088	-0.0092	0.0231	-0.0068	-0.0101	0.0323	-0.0013	0.0073
%PD-1-/CCR7- CD4 T	-0.0127	-0.0092	0.0196	-0.0105	-0.0095	-0.0062	0.0322	-0.0151	0.0073
%CD8 T	0.2259	0.0315	-0.0068	0.0220	-0.0036	0.0206	0.1265	-0.0141	-0.0085
%CD27+ CD8 T	0.0558	-0.0007	0.0334	0.0088	-0.0083	-0.0089	0.0241	-0.0101	0.0191
%CD38+ CD8 T	0.2703	0.0092	0.0742	0.0098	-0.0057	0.0352	0.0711	-0.0026	-0.0173
%CD127+ CD8 T	0.0994	0.0993	-0.0094	-0.0074	-0.0043	0.0017	-0.0059	-0.0076	0.0073
%HLA-DR+ CD8 T	0.0171	0.0221	0.0046	-0.0100	-0.0099	-0.0089	0.0254	-0.0148	-0.0276
%KLRG1+ CD8 T	0.2143	-0.0082	0.0556	0.0043	-0.0066	-0.0038	-0.0018	-0.0176	0.0461
%PD-1+ CD8 T	0.2452	0.0167	0.0147	0.0066	-0.0055	-0.0046	-0.0046	-0.0210	0.0797
%Tn CD8 T	0.2965	-0.0061	0.1125	0.0100	-0.0072	-0.0072	0.0090	-0.0045	0.0315
%Teff CD8 T	-0.0162	-0.0100	0.0196	0.0225	0.0150	-0.0101	-0.0041	-0.0259	0.0091
%Tem CD8 T	0.2719	-0.0073	-0.0075	-0.0061	-0.0018	-0.0076	0.0123	-0.0188	0.2507
%CD38-/HLA-DR+ CD8 T	0.0079	-0.0057	0.0350	-0.0101	-0.0103	-0.0098	-0.0038	0.0053	-0.0255
%CD38+/HLA-DR+ CD8 T	0.0464	0.0422	-0.0096	-0.0080	-0.0093	-0.0028	0.0427	-0.0291	-0.0225
%CD38+/HLA-DR- CD8 T	0.3133	-0.0071	0.0917	0.0274	-0.0026	0.0258	0.0206	0.0081	-0.0019
%KLRG1-/CD27+ CD8 T	0.2091	-0.0082	0.0498	0.0081	-0.0063	-0.0036	0.0039	-0.0152	0.0423
%KLRG1+/CD27+ CD8 T	0.3275	0.0095	0.0035	-0.0069	-0.0067	0.0184	0.0008	-0.0190	0.2280
%KLRG1+/CD27- CD8 T	0.0342	-0.0029	0.0412	0.0056	-0.0091	-0.0090	0.0140	-0.0143	-0.0055
%KLRG1-/CD27- CD8 T	0.0784	-0.0077	-0.0008	-0.0047	-0.0087	-0.0096	0.0178	-0.0242	0.0830
%KLRG1-/PD-1+ CD8 T	-0.0004	0.0190	-0.0020	-0.0081	-0.0066	-0.0081	-0.0101	-0.0291	0.0330
%KLRG1+/PD-1+ CD8 T	0.3072	0.0040	0.0091	0.0055	-0.0064	-0.0055	-0.0022	-0.0199	0.1476
%KLRG1+/PD-1- CD8 T	-0.0333	-0.0012	0.0441	-0.0084	-0.0037	-0.0079	-0.0088	-0.0253	-0.0310
%Tcm CD8 T	0.2214	0.0045	0.0914	-0.0081	0.0087	-0.0059	-0.0035	-0.0220	-0.0179

%KLRG1-/PD-1- CD8 T	0.2653	-0.0051	0.0785	0.0099	-0.0070	-0.0006	-0.0014	-0.0131	0.0347
%PD-1-/CCR7+ CD8 T	0.1951	-0.0049	0.0583	0.0265	-0.0045	-0.0065	0.0041	-0.0107	0.0155
%PD-1+/CCR7+ CD8 T	0.1522	0.0336	0.0400	0.0051	-0.0049	0.0113	0.0122	-0.0255	-0.0152
%PD-1+/CCR7- CD8 T	0.2403	-0.0030	-0.0054	-0.0013	-0.0074	-0.0074	0.0237	-0.0210	0.1407
%PD-1-/CCR7- CD8 T	-0.0454	-0.0028	0.0204	0.0024	0.0066	-0.0109	-0.0040	-0.0212	-0.0134
% NK	0.1107	-0.0083	-0.0085	0.1207	-0.0054	0.0073	-0.0086	-0.0253	-0.0060
%NKG2A+ NK	0.0942	0.0044	-0.0062	0.1548	-0.0087	-0.0072	0.0137	-0.0273	-0.0137
%NKG2A-/CD16+ NK	0.0842	0.0300	-0.0083	0.1167	-0.0092	0.0051	0.0283	-0.0278	-0.0092
%NKG2A+/CD16+ NK	-0.0026	-0.0104	0.0060	0.0601	-0.0102	-0.0098	-0.0103	-0.0262	-0.0294
%NKG2A+/CD16- NK	0.1266	0.0246	-0.0084	0.1358	-0.0060	-0.0001	0.0488	-0.0259	0.0025
%NKG2A-/CD16- NK	-0.0232	0.0279	-0.0089	-0.0059	-0.0104	0.0185	0.0020	-0.0312	-0.0279
% NKT	-0.0088	-0.0007	0.0024	-0.0104	-0.0104	-0.0101	0.0002	-0.0285	0.0641
%NKG2A+ NKT	-0.0352	-0.0106	-0.0107	0.0026	0.0216	-0.0104	-0.0093	-0.0313	-0.0180
%NKG2A-/CD16+ NKT	0.0736	0.1239	-0.0076	0.0219	-0.0087	0.0019	-0.0061	-0.0281	-0.0046
%NKG2A+/CD16+ NKT	0.0377	0.0072	-0.0100	0.0558	0.0081	-0.0078	0.0143	0.0130	0.0013
%NKG2A+/CD16- NKT	-0.0417	-0.0108	-0.0108	-0.0042	0.0180	-0.0101	-0.0065	-0.0317	-0.0200
%NKG2A-/CD16- NKT	0.0807	0.0598	-0.0082	0.0382	0.0197	-0.0062	-0.0095	-0.0281	0.0111
%CD38-/HLA-DR+ Tcm CD4 T	-0.0175	-0.0041	-0.0095	0.0322	0.0003	0.0052	-0.0102	-0.0230	0.0030
%CD38+/HLA-DR+ Tcm CD4 T	0.0821	0.0163	0.0020	-0.0016	-0.0032	-0.0053	0.0767	-0.0275	-0.0209
%CD38+/HLA-DR- Tcm CD4 T	0.0802	-0.0079	0.0000	0.0000	-0.0096	0.0423	0.0252	-0.0277	0.0702
%CD38-/HLA-DR- Tcm CD4 T	0.1225	-0.0085	0.0037	-0.0091	-0.0088	0.0312	0.0615	-0.0266	0.0469
%PD-1+ Tcm CD4 T	-0.0079	0.0028	-0.0089	-0.0095	-0.0066	0.0097	-0.0009	-0.0302	0.0134
%CD38-/HLA-DR+ Tn CD4 T	-0.0677	-0.0061	-0.0101	0.0111	-0.0082	-0.0011	-0.0093	-0.0233	-0.0203
%CD38+/HLA-DR+ Tn CD4 T	-0.0540	-0.0099	-0.0109	-0.0110	-0.0086	-0.0109	0.0375	-0.0319	-0.0141
%CD38+/HLA-DR- Tn CD4 T	0.0214	0.0031	-0.0102	0.0379	-0.0078	0.0143	0.0196	-0.0272	0.0123
%CD38-/HLA-DR- Tn CD4 T	0.0586	0.0084	-0.0098	0.0424	-0.0082	0.0182	0.0772	-0.0258	-0.0053
%PD-1+ Tn CD4 T	0.0467	0.0491	-0.0099	0.0184	-0.0099	0.0165	-0.0083	-0.0292	0.0387
%CD38-/HLA-DR+ Teff CD4 T	0.0272	-0.0101	0.0198	0.0026	-0.0101	-0.0005	0.0103	-0.0116	0.0372
%CD38+/HLA-DR+ Teff CD4 T	0.1986	0.0143	0.0492	-0.0079	0.0035	0.0162	0.0636	-0.0224	0.0374
%CD38+/HLA-DR- Teff CD4 T	0.1137	-0.0090	0.0429	-0.0071	-0.0092	-0.0090	0.0052	0.0009	0.0979
%CD38-/HLA-DR- Teff CD4 T	0.1298	0.0006	0.0432	-0.0085	-0.0038	0.0201	0.0284	-0.0266	0.0369
%PD-1+ Teff CD4 T	0.0117	-0.0058	-0.0057	0.0574	0.0128	-0.0058	0.0030	-0.0228	-0.0141
%CD38-/HLA-DR+ Tem CD4 T	0.0289	0.0205	-0.0024	0.0059	0.0088	0.0131	0.0100	0.0113	0.0171
%CD38+/HLA-DR+ Tem CD4 T	0.1995	0.2090	-0.0082	0.0038	-0.0067	-0.0076	0.0135	-0.0242	-0.0069
%CD38+/HLA-DR- Tem CD4 T	-0.0349	-0.0096	-0.0079	-0.0091	-0.0108	0.0346	0.0205	0.0004	-0.0311
%CD38-/HLA-DR- Tem CD4 T	0.1010	0.1337	-0.0040	0.0098	-0.0038	-0.0069	-0.0073	-0.0225	0.0213
%PD-1+ Tem CD4 T	-0.1078	-0.0082	-0.0037	-0.0110	-0.0098	-0.0115	-0.0115	-0.0339	-0.0261
%CD38-/HLA-DR+ Tcm CD8 T	-0.0815	-0.0049	-0.0080	-0.0081	-0.0098	-0.0108	-0.0077	-0.0319	-0.0244
%CD38+/HLA-DR+ Tcm CD8 T	0.1044	0.0684	0.0211	-0.0048	-0.0076	0.0011	0.0311	-0.0231	-0.0173
%CD38+/HLA-DR- Tcm CD8 T	0.0941	0.0004	0.0356	0.0058	-0.0094	0.0575	0.0310	-0.0242	-0.0261
%CD38-/HLA-DR- Tcm CD8 T	0.1129	0.0431	0.0318	-0.0059	-0.0092	0.0097	0.0395	-0.0257	-0.0267
%PD-1+ Tcm CD8 T	-0.0122	0.0017	-0.0069	0.0008	-0.0094	-0.0105	0.0067	-0.0310	-0.0108
%CD38-/HLA-DR+ Tn CD8 T	0.0043	-0.0012	0.0267	-0.0018	-0.0102	0.0061	-0.0080	-0.0279	-0.0247
%CD38+/HLA-DR+ Tn CD8 T	0.0255	0.0659	-0.0027	-0.0084	-0.0016	-0.0064	0.0194	-0.0298	-0.0256
%CD38+/HLA-DR- Tn CD8 T	0.2663	-0.0007	0.0226	0.0331	-0.0027	0.0361	0.0704	-0.0198	0.0304
%CD38-/HLA-DR- Tn CD8 T	0.2331	-0.0066	0.0105	0.0186	-0.0055	0.0280	0.1271	-0.0224	0.0265
%PD-1+ Tn CD8 T	0.0427	0.0203	0.0282	0.0225	-0.0098	-0.0017	-0.0028	-0.0286	-0.0213
%CD38-/HLA-DR+ Teff CD8 T	0.0641	-0.0072	0.0038	-0.0047	0.0135	-0.0087	-0.0087	0.0001	0.0294
%CD38+/HLA-DR+ Teff CD8 T	0.0110	0.0149	-0.0041	0.0009	-0.0099	-0.0027	0.0329	-0.0288	-0.0059
%CD38+/HLA-DR- Teff CD8 T	0.1132	0.0047	0.0082	-0.0068	0.0047	0.0101	0.0167	0.0052	0.0250
%CD38-/HLA-DR- Teff CD8 T	0.0174	0.0143	-0.0076	0.0188	-0.0067	0.0074	0.0742	-0.0300	-0.0289
%PD-1+ Teff CD8 T	0.0040	-0.0088	-0.0069	-0.0085	-0.0093	-0.0100	0.0010	-0.0264	0.0573
%CD38-/HLA-DR+ Tem CD8 T	-0.0032	-0.0103	0.0026	-0.0010	-0.0027	-0.0097	-0.0104	0.0047	-0.0138
%CD38+/HLA-DR+ Tem CD8 T	0.0310	0.0722	-0.0042	-0.0098	-0.0066	-0.0081	0.0253	-0.0295	-0.0280
%CD38+/HLA-DR- Tem CD8 T	0.0251	-0.0098	-0.0067	-0.0101	0.0021	-0.0034	0.0204	-0.0021	-0.0033
%CD38-/HLA-DR- Tem CD8 T	-0.0031	0.0386	-0.0104	-0.0062	-0.0058	-0.0026	0.0343	-0.0237	-0.0234
%PD-1+ Tem CD8 T	-0.0347	0.0193	-0.0098	0.0059	-0.0106	-0.0104	-0.0103	-0.0317	-0.0171
%IL-2+ CD8 T	-0.0207	0.0277	-0.0100	-0.0077	0.0064	-0.0098	-0.0081	-0.0274	-0.0024

%IL-2+ CD4 T	0.0228	0.0403	-0.0076	0.0109	0.0046	-0.0080	-0.0091	-0.0078	-0.0203
%TNFa+ CD4 T	-0.0307	-0.0063	-0.0103	0.0120	0.0197	-0.0083	0.0039	-0.0104	-0.0183
%TNFa+ CD8 T	-0.0743	-0.0064	-0.0072	-0.0061	0.0091	0.0008	0.0087	-0.0278	-0.0323
%IFNg+ CD4 T	-0.0703	-0.0060	-0.0092	-0.0047	-0.0097	-0.0104	-0.0061	-0.0230	-0.0165
%IFNg+ CD8 T	-0.0946	-0.0096	-0.0107	-0.0102	-0.0065	-0.0084	0.0065	-0.0270	-0.0252
%CD107+ CD4 T	-0.0808	-0.0104	-0.0076	-0.0113	-0.0041	-0.0106	0.0089	-0.0255	-0.0268
%CD107+ CD8 T	-0.0976	-0.0112	-0.0095	-0.0053	-0.0041	-0.0069	0.0060	-0.0335	-0.0322
%CD107+IFNg+IL-2-TNFa- CD4 T	-0.0843	-0.0113	-0.0071	-0.0100	-0.0101	-0.0112	0.0000	-0.0267	-0.0263
%CD107+IFNg-IL-2+TNF- CD4 T	-0.0663	-0.0026	-0.0104	-0.0109	-0.0104	-0.0082	0.0010	-0.0136	-0.0260
%CD107+IFNg-IL-2-TNFa+ CD4 T	-0.0674	-0.0020	-0.0111	-0.0098	0.0072	0.0152	-0.0012	-0.0144	-0.0295
%CD107+IFNg-IL-2-TNFa- CD4 T	-0.0425	-0.0103	-0.0017	-0.0104	0.0125	-0.0101	0.0360	-0.0318	-0.0245
%CD107-IFNg+IL-2+TNFa- CD4 T	-0.0007	0.0366	0.0012	-0.0003	-0.0091	-0.0088	0.0181	-0.0210	-0.0172
%CD107-IFNg+IL-2-TNFa+ CD4 T	-0.0467	-0.0098	-0.0043	0.0321	-0.0071	-0.0027	-0.0007	-0.0319	-0.0240
%CD107-IFNg+IL-2-TNFa- CD4 T	-0.0123	-0.0084	-0.0094	0.0095	-0.0029	-0.0105	-0.0013	-0.0309	-0.0085
%CD107-IFNg-IL-2+TNFa+ CD4 T	0.1650	0.0176	-0.0047	0.0371	0.0844	0.0012	0.0011	-0.0069	-0.0218
%CD107-IFNg-IL-2+TNFa- CD4 T	0.0343	0.0558	-0.0033	0.0125	-0.0101	-0.0037	-0.0074	-0.0144	-0.0231
%CD107-IFNg-IL-2-TNFa+ CD4 T	-0.0145	-0.0025	-0.0100	0.0061	0.0416	-0.0017	-0.0028	-0.0099	-0.0158
%CD107+IFNg+IL-2-TNFa- CD8T	-0.0959	-0.0114	-0.0114	-0.0111	-0.0113	-0.0109	0.0037	-0.0294	-0.0233
%CD107+IFNg-IL-2+TNF- CD8 T	-0.0124	0.0022	0.0118	-0.0105	0.0111	-0.0030	-0.0096	-0.0272	-0.0069
%CD107+IFNg-IL-2-TNFa+ CD8 T	-0.0303	-0.0059	-0.0016	0.0103	-0.0005	0.0175	-0.0011	-0.0303	-0.0198
%CD107+IFNg-IL-2-TNFa- CD8 T	-0.0415	-0.0090	-0.0072	0.0084	0.0022	-0.0081	0.0035	-0.0151	-0.0298
%CD107-IFNg+IL-2+TNFa- CD8 T	-0.0309	0.0060	0.0086	-0.0063	0.0005	-0.0087	-0.0003	-0.0196	-0.0055
%CD107-IFNg+IL-2-TNFa+ CD8 T	-0.0086	-0.0086	0.0011	-0.0094	0.0433	-0.0016	0.0242	-0.0289	-0.0285
%CD107-IFNg+IL-2-TNFa- CD8 T	-0.0450	-0.0021	-0.0109	-0.0104	-0.0083	-0.0106	0.0027	-0.0287	0.0147
%CD107-IFNg-IL-2+TNFa+ CD8 T	0.0301	-0.0083	-0.0065	0.0088	0.0963	0.0004	0.0166	-0.0265	-0.0053
%CD107-IFNg-IL-2+TNFa- CD8 T	-0.0049	0.0488	-0.0099	-0.0094	-0.0104	-0.0097	-0.0096	-0.0287	-0.0089
%CD107-IFNg-IL-2-TNFa+ CD8 T	0.0054	0.0108	-0.0089	-0.0097	0.0188	-0.0093	-0.0102	-0.0214	-0.0187

Supplementary File 1h. *Adjusted R^2 scores and differences in adjusted R^2 for LOCO analysis for the model that contains percent intact.*
The first column contains all immunophenotypes and is a target for the regression model. The second column contains adjusted R^2 of the linear regression model with features such as percent intact, age, biological sex, race, years of treatment, CD4 nadir, recent CD4 count, and years of HIV before treatment ($=$NA, < 1, ≥ 1). The next seven columns show differences in adjusted R^2 score (ΔR^2) after removing a specific feature. Participants with missing years of ART values are excluded from this analysis. The missing value of the CD4 nadir for one participant is imputed.

	Adj. R^2	ΔR^2, difference in adjustable R^2 after dropping a covariate							
Model / Immunophenotype	Include all	% intact HIV DNA	Age	Sex	Years of ART	CD4 nadir	Recent CD4 count	Years before ART	Race
%CD4 T	0.2110	-0.0030	-0.0037	0.0187	0.0119	0.0220	0.1593	-0.0141	-0.0116
%CD27+ CD4 T	0.1079	0.0098	0.0032	0.0209	-0.0054	0.0002	0.0401	-0.0046	0.0630
%CD38+ CD4 T	0.1428	0.0447	0.0499	0.0169	-0.0072	0.0137	-0.0031	-0.0260	0.0174
%CD127+ CD4 T	0.0716	0.0888	0.0268	0.0014	-0.0004	-0.0096	0.0059	-0.0159	-0.0171
%HLA-DR+ CD4 T	0.0959	-0.0087	-0.0057	0.0532	-0.0093	0.0034	0.0388	-0.0066	0.0241
%KLRG1+ CD4 T	0.0783	0.0101	-0.0056	-0.0061	-0.0095	-0.0057	0.0054	-0.0013	0.0253
%NKG2A+ CD4 T	0.0201	-0.0061	0.0138	0.0068	-0.0092	0.0105	0.0027	-0.0164	-0.0055
%PD-1+ CD4 T	0.1005	0.0134	0.0105	0.0206	-0.0062	-0.0003	0.0337	-0.0275	0.0010
%Tcm CD4 T	0.0193	-0.0100	0.0202	-0.0079	-0.0082	-0.0004	-0.0101	-0.0129	-0.0192
%Tn CD4 T	0.0930	0.0014	0.0447	0.0126	-0.0083	-0.0081	0.0349	-0.0277	-0.0169
%Teff CD4 T	-0.0314	-0.0029	-0.0049	-0.0021	-0.0081	0.0025	-0.0004	-0.0220	0.0088
%Tem CD4 T	0.0634	0.0095	-0.0043	0.0037	-0.0096	-0.0092	0.0593	-0.0121	0.0118
%CD38-/HLA-DR+ CD4 T	0.1082	-0.0083	0.0094	0.0650	-0.0027	0.0133	0.0016	0.0126	0.0426
%CD38+/HLA-DR+ CD4 T	0.0474	-0.0030	-0.0085	0.0154	0.0022	-0.0081	0.0714	-0.0268	-0.0129
%CD38+/HLA-DR- CD4 T	0.1674	0.0317	0.0444	0.0388	-0.0087	0.0164	-0.0081	-0.0243	0.0350
%KLRG1-/CD27+ CD4 T	0.0690	0.0162	-0.0066	-0.0096	-0.0093	-0.0033	0.0265	-0.0100	-0.0133
%KLRG1+/CD27+ CD4 T	0.2192	-0.0037	-0.0062	0.0233	-0.0062	0.0366	-0.0080	-0.0234	0.2033
%KLRG1+/CD27- CD4 T	0.0552	0.0101	0.0080	0.0028	-0.0090	0.0077	0.0156	0.0134	0.0068
%KLRG1-/CD27- CD4 T	0.1606	-0.0081	-0.0077	0.0316	0.0021	-0.0040	0.0390	-0.0143	0.1081
%KLRG1-/PD-1+ CD4 T	0.0408	-0.0092	0.0046	0.0078	-0.0084	-0.0006	0.0106	-0.0032	0.0417
%KLRG1+/PD-1+ CD4 T	0.0291	0.0166	-0.0078	-0.0041	-0.0092	-0.0099	0.0025	0.0010	-0.0208
%KLRG1+/PD-1- CD4 T	0.1291	-0.0047	-0.0054	0.0357	-0.0057	0.0014	0.0000	-0.0173	0.0951
%KLRG1-/PD-1- CD4 T	0.1413	0.0188	0.0152	-0.0073	-0.0086	0.0100	0.0456	-0.0242	-0.0250
%PD-1-/CCR7+ CD4 T	0.1133	0.0168	0.0212	0.0138	-0.0080	-0.0057	0.0513	-0.0267	0.0129
%PD-1+/CCR7+ CD4 T	0.0476	-0.0057	0.0153	-0.0044	-0.0057	0.0193	-0.0057	-0.0094	-0.0206
%PD-1+/CCR7- CD4 T	0.0502	0.0109	-0.0085	0.0177	-0.0097	-0.0084	0.0405	-0.0118	0.0038
%PD-1-/CCR7- CD4 T	-0.0034	0.0000	0.0218	-0.0103	-0.0068	-0.0020	0.0389	-0.0209	0.0045
%CD8 T	0.1876	-0.0067	-0.0058	0.0155	-0.0005	0.0192	0.1499	-0.0159	-0.0113
%CD27+ CD8 T	0.0466	-0.0099	0.0303	0.0067	-0.0079	-0.0092	0.0195	-0.0103	0.0142
%CD38+ CD8 T	0.2689	0.0078	0.0702	0.0041	-0.0076	0.0222	0.0787	-0.0088	-0.0163
%CD127+ CD8 T	0.0614	0.0614	-0.0091	-0.0004	0.0194	-0.0077	-0.0016	0.0034	0.0164
%HLA-DR+ CD8 T	-0.0054	-0.0004	0.0078	-0.0089	-0.0069	-0.0102	0.0340	-0.0130	-0.0257
%KLRG1+ CD8 T	0.2160	-0.0064	0.0557	0.0054	-0.0076	-0.0026	-0.0021	-0.0163	0.0425
%PD-1+ CD8 T	0.2212	-0.0072	0.0185	0.0096	-0.0037	-0.0049	-0.0017	-0.0220	0.0786
%Tn CD8 T	0.2964	-0.0063	0.1148	0.0114	-0.0073	-0.0071	0.0100	-0.0039	0.0309
%Teff CD8 T	-0.0163	-0.0101	0.0189	0.0226	0.0111	-0.0103	-0.0048	-0.0252	0.0102
%Tem CD8 T	0.2718	-0.0074	-0.0075	-0.0062	-0.0018	-0.0075	0.0116	-0.0184	0.2452
%CD38-/HLA-DR+ CD8 T	0.0034	-0.0102	0.0376	-0.0103	-0.0102	-0.0098	-0.0021	0.0032	-0.0249
%CD38+/HLA-DR+ CD8 T	0.0175	0.0133	-0.0102	-0.0044	-0.0029	-0.0076	0.0550	-0.0290	-0.0223
%CD38+/HLA-DR- CD8 T	0.3145	-0.0059	0.0932	0.0257	-0.0042	0.0205	0.0198	0.0042	-0.0007
%KLRG1-/CD27+ CD8 T	0.2101	-0.0073	0.0497	0.0089	-0.0071	-0.0028	0.0034	-0.0143	0.0393
%KLRG1+/CD27+ CD8 T	0.3146	-0.0034	0.0055	-0.0064	-0.0071	0.0200	-0.0017	-0.0200	0.2143
%KLRG1+/CD27- CD8 T	0.0270	-0.0101	0.0382	0.0042	-0.0090	-0.0094	0.0103	-0.0139	-0.0088
%KLRG1-/CD27- CD8 T	0.0794	-0.0066	-0.0003	-0.0060	-0.0073	-0.0095	0.0174	-0.0255	0.0810
%KLRG1-/PD-1+ CD8 T	-0.0295	-0.0101	0.0008	-0.0076	-0.0069	-0.0096	-0.0106	-0.0310	0.0404
%KLRG1+/PD-1+ CD8 T	0.2978	-0.0055	0.0113	0.0081	-0.0050	-0.0051	-0.0001	-0.0200	0.1414
%KLRG1+/PD-1- CD8 T	-0.0427	-0.0106	0.0403	-0.0089	-0.0042	-0.0072	-0.0100	-0.0244	-0.0313
%Tcm CD8 T	0.2086	-0.0082	0.0980	-0.0082	0.0093	-0.0065	-0.0056	-0.0229	-0.0179
%KLRG1-/PD-1- CD8 T	0.2640	-0.0063	0.0811	0.0117	-0.0076	0.0002	-0.0004	-0.0125	0.0326

%PD-1-/CCR7+ CD8 T	0.1925	-0.0075	0.0609	0.0288	-0.0063	-0.0062	0.0059	-0.0107	0.0154
%PD-1+/CCR7+ CD8 T	0.1092	-0.0093	0.0479	0.0077	-0.0039	0.0074	0.0038	-0.0271	-0.0202
%PD-1+/CCR7- CD8 T	0.2370	-0.0063	-0.0049	0.0002	-0.0065	-0.0077	0.0271	-0.0207	0.1373
%PD-1-/CCR7- CD8 T	-0.0536	-0.0109	0.0178	0.0011	0.0060	-0.0110	-0.0062	-0.0207	-0.0151
% NK	0.1105	-0.0085	-0.0086	0.1232	-0.0043	0.0078	-0.0087	-0.0258	-0.0073
%NKG2A+ NK	0.0882	-0.0015	-0.0075	0.1503	-0.0075	-0.0092	0.0070	-0.0259	-0.0181
%NKG2A-/CD16+ NK	0.0570	0.0028	-0.0096	0.1143	-0.0083	-0.0034	0.0147	-0.0265	-0.0179
%NKG2A+/CD16+ NK	0.0380	0.0302	0.0055	0.0474	-0.0080	-0.0043	-0.0094	-0.0176	-0.0293
%NKG2A+/CD16- NK	0.0943	-0.0077	-0.0077	0.1467	-0.0086	-0.0004	0.0378	-0.0269	-0.0054
%NKG2A-/CD16- NK	-0.0583	-0.0072	-0.0073	-0.0070	-0.0109	0.0089	-0.0049	-0.0322	-0.0316
% NKT	-0.0004	0.0077	0.0053	-0.0104	-0.0083	-0.0069	0.0066	-0.0305	0.0496
%NKG2A+ NKT	-0.0329	-0.0083	-0.0107	0.0041	0.0134	-0.0107	-0.0094	-0.0308	-0.0168
%NKG2A-/CD16+ NKT	-0.0613	-0.0109	-0.0109	0.0282	-0.0110	-0.0035	-0.0110	-0.0310	-0.0169
%NKG2A+/CD16+ NKT	0.0217	-0.0088	-0.0098	0.0569	0.0070	-0.0093	0.0078	0.0048	0.0043
%NKG2A+/CD16- NKT	-0.0382	-0.0073	-0.0107	-0.0031	0.0101	-0.0107	-0.0071	-0.0318	-0.0183
%NKG2A-/CD16- NKT	0.0118	-0.0092	-0.0100	0.0464	0.0081	-0.0073	-0.0094	-0.0302	0.0046
%CD38-/HLA-DR+ Tcm CD4 T	-0.0226	-0.0093	-0.0090	0.0320	0.0005	0.0012	-0.0094	-0.0255	-0.0004
%CD38+/HLA-DR+ Tcm CD4 T	0.0719	0.0061	-0.0002	0.0033	0.0060	-0.0083	0.0876	-0.0266	-0.0242
%CD38+/HLA-DR- Tcm CD4 T	0.0986	0.0105	0.0016	-0.0025	-0.0077	0.0236	0.0199	-0.0275	0.0589
%CD38-/HLA-DR- Tcm CD4 T	0.1477	0.0167	0.0044	-0.0085	-0.0079	0.0146	0.0587	-0.0236	0.0370
%PD-1+ Tcm CD4 T	-0.0158	-0.0051	-0.0079	-0.0097	-0.0086	0.0023	0.0045	-0.0311	0.0068
%CD38-/HLA-DR+ Tn CD4 T	-0.0586	0.0029	-0.0105	0.0082	-0.0041	-0.0070	-0.0105	-0.0282	-0.0237
%CD38+/HLA-DR+ Tn CD4 T	-0.0486	-0.0045	-0.0109	-0.0107	-0.0105	-0.0101	0.0389	-0.0310	-0.0156
%CD38+/HLA-DR- Tn CD4 T	0.0197	0.0014	-0.0101	0.0349	-0.0097	0.0039	0.0117	-0.0295	0.0029
%CD38-/HLA-DR- Tn CD4 T	0.0445	-0.0056	-0.0097	0.0420	-0.0093	0.0094	0.0639	-0.0282	-0.0120
%PD-1+ Tn CD4 T	-0.0061	-0.0037	-0.0100	0.0194	-0.0103	0.0054	-0.0105	-0.0296	0.0223
%CD38-/HLA-DR+ Teff CD4 T	0.0283	-0.0090	0.0202	0.0015	-0.0099	-0.0027	0.0102	-0.0142	0.0342
%CD38+/HLA-DR+ Teff CD4 T	0.2131	0.0288	0.0458	-0.0081	0.0215	0.0301	0.0699	-0.0178	0.0344
%CD38+/HLA-DR- Teff CD4 T	0.1154	-0.0072	0.0444	-0.0075	-0.0092	-0.0092	0.0040	-0.0037	0.0926
%CD38-/HLA-DR- Teff CD4 T	0.1441	0.0149	0.0415	-0.0065	0.0062	0.0316	0.0307	-0.0252	0.0311
%PD-1+ Teff CD4 T	0.0080	-0.0094	-0.0064	0.0527	0.0149	-0.0054	0.0052	-0.0227	-0.0145
%CD38-/HLA-DR+ Tem CD4 T	0.0130	0.0045	-0.0003	0.0127	-0.0035	0.0193	0.0054	0.0162	0.0199
%CD38+/HLA-DR+ Tem CD4 T	0.0081	0.0175	-0.0098	0.0149	0.0040	-0.0077	0.0333	-0.0303	-0.0089
%CD38+/HLA-DR- Tem CD4 T	-0.0345	-0.0093	-0.0081	-0.0085	-0.0105	0.0353	0.0202	0.0014	-0.0308
%CD38-/HLA-DR- Tem CD4 T	-0.0151	0.0177	-0.0002	0.0223	-0.0106	-0.0047	-0.0022	-0.0236	0.0215
%PD-1+ Tem CD4 T	-0.1108	-0.0111	-0.0045	-0.0107	-0.0090	-0.0115	-0.0116	-0.0340	-0.0273
%CD38-/HLA-DR+ Tcm CD8 T	-0.0865	-0.0099	-0.0087	-0.0070	-0.0109	-0.0111	-0.0064	-0.0319	-0.0226
%CD38+/HLA-DR+ Tcm CD8 T	0.0358	-0.0002	0.0136	-0.0008	-0.0022	-0.0030	0.0470	-0.0263	-0.0244
%CD38+/HLA-DR- Tcm CD8 T	0.0853	-0.0084	0.0398	0.0060	-0.0094	0.0467	0.0247	-0.0259	-0.0265
%CD38-/HLA-DR- Tcm CD8 T	0.0702	0.0004	0.0250	-0.0026	-0.0069	0.0038	0.0533	-0.0278	-0.0280
%PD-1+ Tcm CD8 T	-0.0203	-0.0065	-0.0078	0.0040	-0.0106	-0.0104	0.0103	-0.0311	-0.0141
%CD38-/HLA-DR+ Tn CD8 T	-0.0041	-0.0096	0.0303	-0.0015	-0.0102	0.0023	-0.0061	-0.0292	-0.0269
%CD38+/HLA-DR+ Tn CD8 T	-0.0474	-0.0069	-0.0067	-0.0065	0.0049	-0.0080	0.0339	-0.0320	-0.0311
%CD38+/HLA-DR- Tn CD8 T	0.2610	-0.0061	0.0256	0.0327	-0.0041	0.0278	0.0630	-0.0212	0.0289
%CD38-/HLA-DR- Tn CD8 T	0.2343	-0.0054	0.0101	0.0157	-0.0071	0.0214	0.1311	-0.0229	0.0292
%PD-1+ Tn CD8 T	0.0124	-0.0100	0.0344	0.0251	-0.0102	-0.0044	-0.0070	-0.0301	-0.0259
%CD38-/HLA-DR+ Teff CD8 T	0.0652	-0.0060	0.0033	-0.0031	0.0056	-0.0094	-0.0089	-0.0046	0.0260
%CD38+/HLA-DR+ Teff CD8 T	0.0166	0.0205	-0.0054	-0.0047	-0.0035	-0.0082	0.0387	-0.0301	-0.0058
%CD38+/HLA-DR- Teff CD8 T	0.1050	-0.0035	0.0062	-0.0083	-0.0027	0.0049	0.0213	-0.0001	0.0194
%CD38-/HLA-DR- Teff CD8 T	0.0102	0.0071	-0.0086	0.0104	0.0012	-0.0003	0.0843	-0.0302	-0.0296
%PD-1+ Teff CD8 T	0.0031	-0.0097	-0.0073	-0.0085	-0.0097	-0.0097	0.0030	-0.0256	0.0573
%CD38-/HLA-DR+ Tem CD8 T	-0.0027	-0.0098	0.0026	-0.0004	-0.0047	-0.0093	-0.0104	0.0014	-0.0147
%CD38+/HLA-DR+ Tem CD8 T	-0.0297	0.0115	-0.0076	-0.0080	0.0039	-0.0105	0.0391	-0.0307	-0.0283
%CD38+/HLA-DR- Tem CD8 T	0.0254	-0.0095	-0.0068	-0.0101	-0.0008	-0.0048	0.0212	-0.0048	-0.0044
%CD38-/HLA-DR- Tem CD8 T	-0.0409	0.0007	-0.0105	-0.0094	0.0017	-0.0064	0.0466	-0.0231	-0.0249
%PD-1+ Tem CD8 T	-0.0635	-0.0095	-0.0108	0.0097	-0.0097	-0.0108	-0.0089	-0.0326	-0.0203
%IL-2+ CD8 T	-0.0499	-0.0014	-0.0109	-0.0080	0.0090	-0.0109	-0.0108	-0.0313	0.0079
%IL-2+ CD4 T	-0.0078	0.0097	-0.0093	0.0203	-0.0071	-0.0102	-0.0104	-0.0037	-0.0201

%TNFa+ CD4 T	-0.0314	-0.0069	-0.0101	0.0153	0.0098	-0.0070	0.0028	-0.0084	-0.0184
%TNFa+ CD8 T	-0.0553	0.0127	-0.0081	-0.0077	0.0208	-0.0070	0.0034	-0.0312	-0.0301
%IFNg+ CD4 T	-0.0723	-0.0080	-0.0087	-0.0029	-0.0110	-0.0097	-0.0070	-0.0218	-0.0170
%IFNg+ CD8 T	-0.0793	0.0057	-0.0108	-0.0108	-0.0005	-0.0109	0.0028	-0.0307	-0.0211
%CD107+ CD4 T	-0.0816	-0.0112	-0.0073	-0.0113	-0.0053	-0.0107	0.0080	-0.0260	-0.0269
%CD107+ CD8 T	-0.0859	0.0005	-0.0097	-0.0070	0.0017	-0.0100	0.0037	-0.0330	-0.0306
%CD107+IFNg+IL-2-TNFa- CD4 T	-0.0839	-0.0109	-0.0069	-0.0098	-0.0098	-0.0111	-0.0003	-0.0277	-0.0259
%CD107+IFNg-IL-2+TNF- CD4 T	-0.0692	-0.0055	-0.0107	-0.0103	-0.0111	-0.0098	0.0031	-0.0113	-0.0241
%CD107+IFNg-IL-2-TNFa+ CD4 T	-0.0745	-0.0091	-0.0112	-0.0089	0.0007	0.0160	-0.0032	-0.0141	-0.0293
%CD107+IFNg-IL-2-TNFa- CD4 T	-0.0426	-0.0103	-0.0021	-0.0103	0.0088	-0.0098	0.0393	-0.0319	-0.0243
%CD107-IFNg+IL-2+TNFa- CD4 T	-0.0050	0.0323	-0.0015	-0.0067	0.0021	-0.0104	0.0251	-0.0139	-0.0216
%CD107-IFNg+IL-2-TNFa+ CD4 T	-0.0451	-0.0081	-0.0049	0.0334	-0.0089	-0.0004	0.0015	-0.0320	-0.0256
%CD107-IFNg+IL-2-TNFa- CD4 T	-0.0144	-0.0104	-0.0091	0.0097	-0.0036	-0.0106	0.0005	-0.0309	-0.0070
%CD107-IFNg-IL-2+TNFa+ CD4 T	0.1469	-0.0006	-0.0062	0.0458	0.0550	-0.0027	-0.0022	-0.0051	-0.0221
%CD107-IFNg-IL-2+TNFa- CD4 T	-0.0319	-0.0104	-0.0070	0.0173	-0.0103	-0.0040	-0.0026	-0.0193	-0.0197
%CD107-IFNg-IL-2-TNFa+ CD4 T	-0.0144	-0.0024	-0.0102	0.0020	0.0526	-0.0055	-0.0014	-0.0149	-0.0166
%CD107+IFNg+IL-2-TNFa- CD8T	-0.0858	-0.0013	-0.0113	-0.0113	-0.0098	-0.0113	0.0020	-0.0316	-0.0203
%CD107+IFNg-IL-2+TNF- CD8 T	0.0178	0.0324	0.0137	-0.0094	0.0343	-0.0090	-0.0091	-0.0296	-0.0017
%CD107+IFNg-IL-2-TNFa+ CD8 T	-0.0309	-0.0065	-0.0029	0.0091	0.0018	0.0096	-0.0038	-0.0288	-0.0219
%CD107+IFNg-IL-2-TNFa- CD8 T	-0.0397	-0.0071	-0.0068	0.0056	0.0063	-0.0095	0.0043	-0.0129	-0.0298
%CD107-IFNg+IL-2+TNFa- CD8 T	-0.0143	0.0226	0.0049	-0.0078	0.0114	-0.0105	-0.0058	-0.0274	0.0065
%CD107-IFNg+IL-2-TNFa+ CD8 T	0.0437	0.0436	-0.0001	-0.0099	0.0737	-0.0091	0.0168	-0.0292	-0.0236
%CD107-IFNg+IL-2-TNFa- CD8 T	-0.0468	-0.0039	-0.0109	-0.0106	-0.0063	-0.0091	-0.0015	-0.0310	0.0221
%CD107-IFNg-IL-2+TNFa+ CD8 T	0.0308	-0.0076	-0.0062	0.0110	0.0759	0.0021	0.0162	-0.0255	-0.0052
%CD107-IFNg-IL-2+TNFa- CD8 T	-0.0628	-0.0092	-0.0092	-0.0082	-0.0106	-0.0104	-0.0070	-0.0302	-0.0033
%CD107-IFNg-IL-2-TNFa+ CD8 T	0.0244	0.0298	-0.0081	-0.0101	0.0444	-0.0040	-0.0101	-0.0132	-0.0230

Supplementary File 1i. *Host features classify PWH with respect to HIV reservoir characteristics.*
The frequency of each of the 144 variables was analyzed for performance in classifying each PWH within the cohort as having reservoir frequency above or below the median using a receiver operating characteristics (ROC) curve. The area under the curve (AUC) is reported. The table includes variables that had an AUC value larger than 0.6 for one of the reservoir frequency characteristics. We highlight variables that had an AUC value higher than 0.65 in **bold**. Variables are ranked by AUC for the total reservoir frequency. For years of ART, AUC values are computed based on 108 PWH, excluding participants with missing years of ART values. For CD4 Nadir, AUC values are computed based on 114 PWH, excluding one participant] with the missing CD4 Nadir value.

Variable	Bin Total HIV DNA	Bin Intact HIV DNA	Bin % intact HIV DNA	Variable	Bin Total HIV DNA	Bin Intact HIV DNA	Bin % intact HIV DNA
%NKG2A+ CD4 T	**0.6953**	0.6084	0.5416	%PD-1-/CCR7+ CD8 T	0.6207	0.6134	0.5115
%PD-1+ Tn CD4 T	**0.6827**	0.5749	0.5313	%CD38+ CD8 T	0.6142	0.5425	0.6222
%CD38+/HLA-DR- CD8 T	**0.6777**	0.5797	0.6084	%CD38-/HLA-DR- Teff CD4 T	0.6122	0.5587	0.5017
%Tn CD4 T	**0.6763**	0.5933	0.5665	%CD38-/HLA-DR+ Tn CD8 T	0.6122	0.5420	0.5933
%KLRG1-/PD-1- CD4 T	**0.6745**	0.5789	0.6224	%KLRG1-/CD27+ CD8 T	0.6096	0.6101	0.5265
%Tcm CD8 T	**0.6729**	0.6205	0.5541	%CD38+/HLA-DR- Tn CD4 T	0.6078	0.5050	0.5531
%CD38+/HLA-DR- CD4 T	**0.6700**	0.5507	0.5889	%CD107-IFNγ+IL-2-TNFα- CD8 T	0.6065	0.5712	0.5133
%CD8 T	**0.6629**	0.6547	0.5484	%CD38+/HLA-DR+ Tem CD4 T	0.6057	0.5829	0.5941
Age	**0.6627**	0.6264	0.5476	%CD38+/HLA-DR- Tcm CD8 T	0.6039	0.5150	0.5750
%PD-1+/CCR7+ CD8 T	**0.6608**	0.5907	0.5328	%CD38-/HLA-DR+ Tcm CD8 T	0.6030	0.6053	0.5015
%PD-1+ CD8 T	**0.6562**	0.6207	0.5070	%KLRG1-/PD-1+ CD4 T	0.6019	0.5346	0.5290
%PD-1+ Tn CD8 T	**0.6553**	0.5880	0.5597	%NKG2A-/CD16+ NK	0.6012	0.5257	0.5848
%Tn CD8 T	**0.6550**	0.6485	0.5138	%KLRG1+ CD8 T	0.5954	0.6021	0.5251
%PD-1-/CCR7+ CD4 T	**0.6537**	0.5473	0.6178	%KLRG1-/CD27+ CD4 T	0.5945	0.5322	0.6205
%PD-1+/CCR7+ CD4 T	0.6475	0.5656	0.5740	%CD27+ CD4 T	0.5776	0.5018	0.6021
%Tcm CD4 T	0.6452	0.5998	0.5104	%KLRG1+/CD27- CD4 T	0.5723	0.5017	0.6181
%KLRG1-/PD-1- CD8 T	0.6440	0.6308	0.5234	%PD-1+ Tcm CD4 T	0.5705	0.5005	0.6158
%PD-1+ CD4 T	0.6434	0.5286	0.6295	%PD-1+ Tem CD4 T	0.5688	0.6124	0.5489
%CD4 T	0.6429	0.6338	0.5322	%KLRG1+ CD4 T	0.5649	0.5243	0.6236
%CD107-IFNγ+IL-2-TNFα- CD4 T	0.6420	0.5670	0.5185	Recent CD4 count	0.5620	0.6320	0.6104
%CD38+ CD4 T	0.6414	0.5299	0.5932	%KLRG1+/PD-1+ CD4 T	0.5602	0.5100	0.6450
%CD127+ CD4 T	0.6367	**0.7051**	0.6323	%Tem CD4 T	0.5576	0.5230	0.6145
%CD38+/HLA-DR- Tn CD8 T	0.6364	0.5558	0.5724	%IL-2+ CD4 T	0.5416	0.6022	0.5687
%PD-1-/CCR7- CD4 T	0.6340	0.6010	0.5473	%CD107-IFNγ+IL-2+TNFα- CD4 T	0.5342	0.6308	0.6248
%KLRG1+/PD-1+ CD8 T	0.6335	0.6098	0.5020	%CD38+/HLA-DR- Teff CD8 T	0.5306	0.5613	0.6276
CD4 Nadir	0.6303	0.6110	0.5563	Years before ART < 1	0.5299	0.5222	0.6092
Years of ART	0.6296	0.5069	**0.7171**	%CD127+ CD8 T	0.5195	0.6051	0.6048
%CD38-/HLA-DR- Tem CD4 T	0.6249	0.5513	0.5867	%CD107-IFNγ-IL-2+TNFα+ CD4 T	0.5003	0.6000	0.6493
%KLRG1+/CD27+ CD8 T	0.6224	0.6473	0.5153				

Supplementary File 1j Training procedure for classification (regression)

Input: Outcome, algorithm, set of hyperparameters from Supplementary File 1k
Output: Mean and standard deviation of train and test accuracies (R^2 scores)

Compute product of hyperparameters for grid search, let H be one hyperparameter setting
for k in the set of top AUC (correlation) variables to consider **do**
    Take the first k variables from the set of variables
    for test seed in the array of random seeds for train/test splits **do**
        Sort data according to test seed
        Split data into test and train in proportion 25% to 75%
        for every hyperparameters settings H **do**
            for validation seed in the array of random seeds for train/valid splits **do**
                Sort data according to validation seed
                Split data into validation and train for validation in proportion 25% to 75%
                Train algorithm with hyperparameter setting H on train for the validation set
                Compute accuracy (R^2 score) on validation set
            end for
            Compute mean validation accuracy (R^2 score) on all validation sets for hyperparameter setting H
        end for
        Find hyperparameter setting (H_m) with the highest mean validation accuracy
        Train algorithm with hyperparameter setting H_m on train data
        Compute train and test accuracy (R^2 score)
    end for
    Compute accuracy (R^2 score) mean and std over different train and test sorting
end for
Among mean validation accuracies (R^2 scores) for different k find the maximum
Return train and test accuracies (R^2 scores) mean and standard deviation that corresponds to the number of variables k with the highest mean validation accuracy (R^2 score)

Supplementary File 1k. *Ranges of hyperparameters values that we used to perform grid search for classification and regression.*

**General**	
Random seeds for train/test splits	0..10
Random seeds for train/valid splits	0..20
Set of the top AUC variables to consider	2..9,10,15,20,25,30,40,50
Set of the top absolute value of correlation coefficient to consider	2..9,10,15,20,25,30,40, all
**Logistic Regression** (classifier)	
Penalty	L2
Regularization parameter	0.00001, 0.0001, 0.001, 0.01, 0.1, 1, 10, 100, 1000, 10000
**SVM with RBF kernel** (classifier)	
Regularization parameter	0.00001, 0.0001, 0.001, 0.01, 0.1, 1, 10, 100, 1000, 10000
**Ridge Regression** (regressor)	
Regularization parameter	0.00001, 0.0001, 0.001, 0.01, 0.1, 1, 10, 100, 1000, 10000
**Kernel Ridge Regression** (regressor)	
Regularization parameter	0.00001, 0.0001, 0.001, 0.01, 0.1, 1, 10, 100, 1000, 10000
Kernel parameter	0.00001, 0.0001, 0.001, 0.01, 0.1, 1, 10, 100, 1000, 10000
**CART** (classifier) and **Decition Tree** (regressor)	
Maximum depth	2..7, 10
Minimum number of samples in the leaf	4, 6, 8, 10
**Random Forest and Gradient Boosted Trees** (classifier and regressor)	
Maximum depth	2..7, 10
Minimum number of samples in the leaf	4, 6, 8, 10
Maximum number of estimators	2, 5, 10, 20, 50, 100, 200
